# A new method for predicting SIRS after percutaneous transhepatic gallbladder drainage

**DOI:** 10.1038/s41598-023-48908-6

**Published:** 2023-12-06

**Authors:** Xuanfeng Zhang, Lulu Yang, Long Cui, Huansong Li, Xiaochuan Wang

**Affiliations:** 1 Center of Hepatobiliary Pancreatic Disease, XuZhou Central Hospital, No.199 Jiefang South Road, Xuzhou, Jiangsu People’s Republic of China; 2 Department of Radiology, XuZhou Central Hospital, Xuzhou, Jiangsu People’s Republic of China

**Keywords:** Cholecystitis, Cholelithiasis

## Abstract

The occurrence of systemic inflammatory response after percutaneous transhepatic gallbladder drainage brings great risks to patients and is one of the challenges faced by clinicians. Therefore, it is of great significance to find a suitable prediction method for clinicians to intervene early and reduce the transformation of serious complications. Easy-to-obtain and objectively measured clinical features were screened, and logistic regression was used to construct a prediction model. The predictive ability of the model was evaluated by using the receiver operating characteristic curve and the decision curve in the validation set and the training set, respectively. Nine clinical features (CRP, Fever, DBIL, Obstruction, Bile properties, PCT, Length, Width, and Volume factor) were used to construct the prediction model, and the validation results showed that the prediction model had good performance in the training set (AUC = 0.83) and the validation set (AUC = 0.81). The decision curve also showed that the predictive ability of the model incorporating nine clinical features is better than that of a single clinical feature. The model we constructed can accurately predict the occurrence of SIRS, which can guide clinicians to take treatment measures and avoid the deterioration of complications.

## Introduction

Previous studies have shown that about 10–18% of patients with asymptomatic gallstone will have biliary pain, which will recur throughout their lives^1^. Among them, 1–4% of patients will develop more serious complications, such as acute cholecystitis (AC), gallstone pancreatitis and choledocholithiasis^2^. Gallstones move in the gallbladder, sometimes stopping the excretion of bile and causing inflammation and infection of the gallbladder, which can lead to persistent and severe abdominal pain, fever, nausea and vomiting^3^. As the condition progresses, AC may result in a chronic blockage of the cystic duct, increased gallbladder pressure from ongoing mucus production, venous bleeding followed by arterial hemorrhage, and ischemic gallbladder necrosis. Necrotic tissue subsequently causes complications such as gallbladder perforation, pulmonary edema, and systemic inflammatory response syndrome (SIRS)^4^.

Studies have shown that the incidence of AC is positively correlated with age, which seriously affects the health and quality of life of the elderly population, especially those who cannot undergo cholecystectomy due to physical conditions^5^. The mortality rate for both open and laparoscopic cholecystectomy is typically less than 0.5%. The mortality rate following cholecystectomy is significantly higher in individuals with high surgical risk, it can reach 10% in older patients and those with internal medical comorbidities^6^. Percutaneous transhepatic gallbladder drainage (PTGD) is a minimally invasive, safe, and successful nonsurgical treatment, especially appropriate for patients with AC at high surgical risk because these patients have a poor prognosis and are at high risk of undergoing radical cholecystectomy^7^. PTGD can relieve gallbladder pressure and cholecystitis symptoms in a timely manner, avoid a series of complications related to AC, and help AC patients with high surgical risks survive the acute crisis period^8^.

Is PTGD completely safe? of course not. The patient's comfort and quality of life will suffer from long-term biliary drainage, which will also raise the risk of complications and readmission^9^. As an invasive procedure, PTGD has consequences that affect 3–13% of treated individuals and include biliary peritonitis, severe bleeding, hemothorax, and pneumothorax^4^. SIRS is a frequent occurrence during recovery after PTGD. The existence of SIRS 72 h after percutaneous cholecystostomy was found to increase 30-day mortality in AC patients, and may serve as an indication of the effectiveness and prognosis of treatment^10^. The persistence of SIRS will further induce multiple organ dysfunction syndrome (MODS), especially the disorders of the circulatory system and respiratory system, which further increase the mortality rate of patients and the probability of intensive care intervention^11^.

Therefore, finding a sensitive predictive method for the occurrence of SIRS after PTGD is of great significance for guiding the clinical treatment of AC patients. Early prediction of the occurrence of SIRS and timely intensive care treatment are valuable for reducing the mortality of AC patients.

## Methods

### Patients

This study comprised 129 adult patients who had PTGD therapy at Xuzhou Central Hospital between January 2020 and December 2022.

Diagnosis of AC and SIRS.

Local inflammatory signs, such as Murphy's sign or a mass, pain, or tenderness in the right upper quadrant; systemic inflammatory symptoms, such as fever, an elevated C-reactive protein (CRP), and an elevated white blood cell count (WBC); and imaging findings specific to AC were used to identify AC. Local inflammation/upper abdominal symptoms and systemic inflammation combined with imaging findings can definitively diagnose AC.

SIRS can be considered if ≥ 2 of the following indicators are met, 1. Body temperature > 38 °C or < 36 °C; 2, heart rate > 90 beats/min; 3. If the respiratory rate is greater than 20 times/min, or the partial pressure of carbon dioxide in arterial blood is less than 32 MMHG during blood gas examination; 4, if the white blood cell count > 12 × 10^9/L, or < 4 × 10^9/L, and immature granulocytes > 10%.

### Ethical approval

This study is a retrospective study and has been approved by the Ethics Committee of Xuzhou Central Hospital. The clinical information and imaging data involved in the study have obtained the informed consent of all participants and/or their Legal guardian. All methods were performed in accordance with the relevant guidelines and regulations.

### Definition of special clinical characteristics

**Figure 1:**
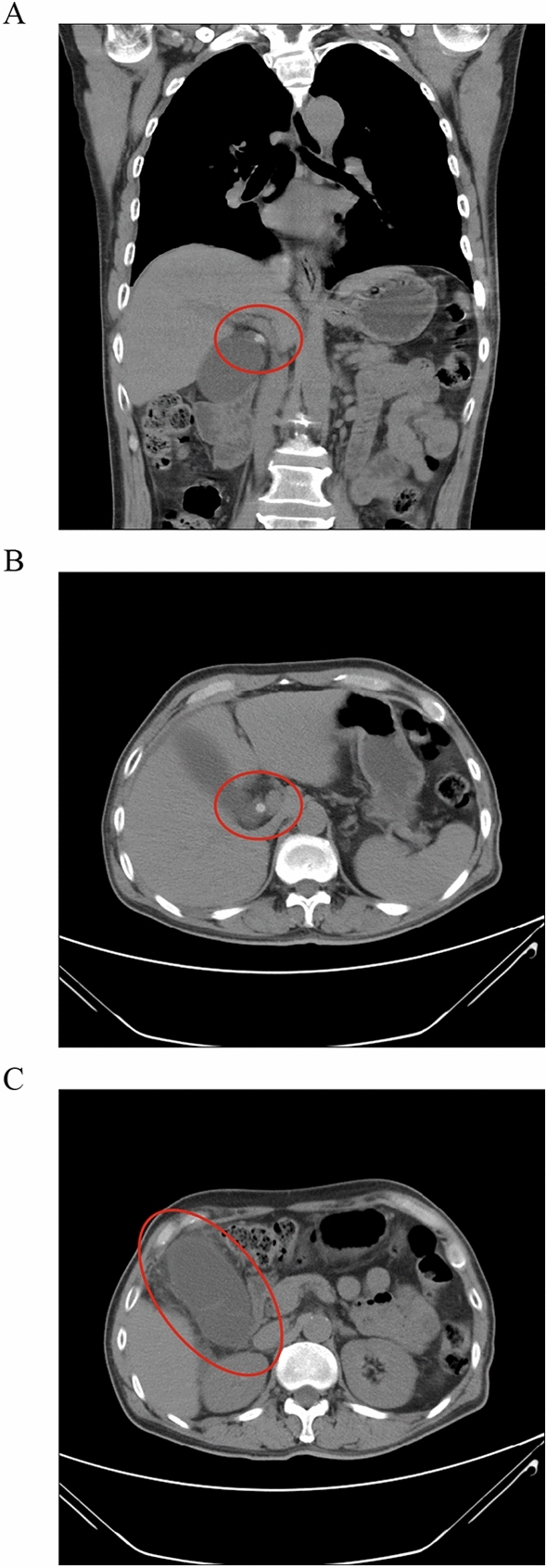
Interpretation of specific clinical features. (**A**) CT scan showed obstruction of stones in the cystic duct (coronal view); (**B**) CT scan showed obstruction of stones in the cystic duct (axial view); (**C**) CT scan showed inflammatory reaction around the gallbladder.

**Figure 2:**
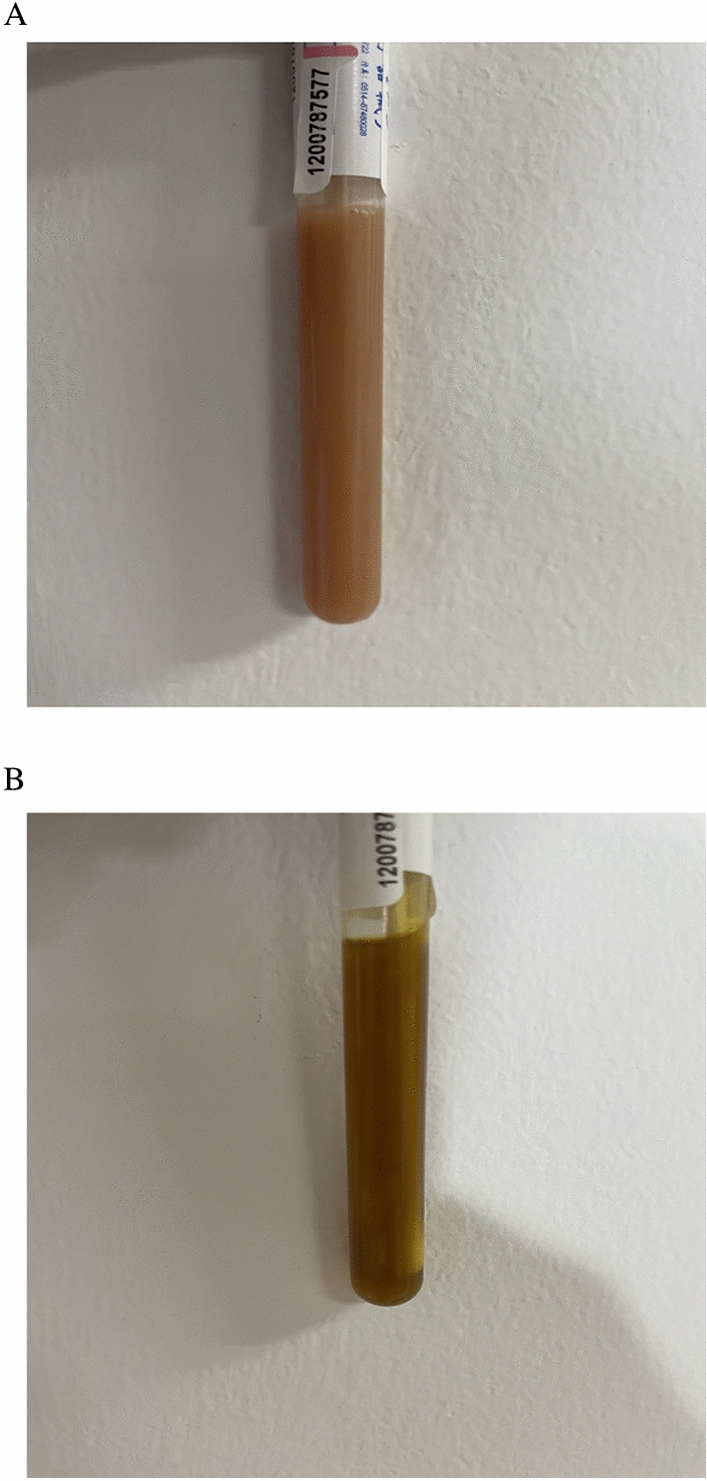
Illustration of bile properties after PTGD. (**A**) Purulent bile; (**B**) Normal bile.

In view of the need to build a clinical prediction model with broad applicability, we screened and used objective clinical characteristics (clinical features that can be objectively measured). Limited by text length, each clinical feature was simplified and abbreviated, and the corresponding search results for each clinical feature can be found in Table 1. For clinical features that cannot be objectively measured, we provide a further explanation here. After CT examination is completed, a cystic duct obstruction is found, which can be defined as a cystic duct obstruction (Obstruction) (Fig. 1A and B). CT images show fuzzy exudation around the gallbladder, which can be defined as Inflammatory exudation (inflammatory reaction around the gallbladder) (Fig. 1C). After PTGD, bile in the gallbladder is extracted by syringe, which can be divided into purulent bile (Fig. 2A) and normal bile (Fig. 2B) according to its properties.

**Table 1:** Interpretation of the clinical features involved in the study.

clinical characteristics	Interpretation of clinical characteristics
Blood bacterial	Peripheral blood bacterial culture results
Bile bacteria	Bile bacterial culture results
Abdominal pain time	Abdominal pain time before admission (day)
WBC	WBC level at the time of hospitalization (*10^9/L)
CRP	CRP level at the time of hospitalization (mg/L)
DM	Diabetes mellitus level at the time of hospitalization
Fever	Fever before admission
DBIL	The direct bilirubin value of the first blood draw after admission (umol/L)
Obstruction	CT or MRI suggests cystic duct obstruction
Bile properties	Characteristics of bile after percutaneous gallbladder drainage
Inflammatory exudation	CT scan shows inflammatory reaction around the gallbladder
PCT	Procalcitonin level at the time of hospitalization
Length	Gallbladder length (mm)
Width	Gallbladder width (mm)
Volume factor	Gallbladder length * Gallbladder width
Wall thickness	Gallbladder wall thickness (mm)

### Statistical analysis

Means and standard deviations were shown for all numerical variables. The χ2 test was used to compare categorical variables. Statistics were deemed significant at *p* < 0.05. All statistical analyses were performed using R software (version 4.2.0).

### Ethical approval and consent to participate

The Xuzhou Central Hospital's ethical committee gave its approval for this study. 

### Informed consent

Informed consent was obtained from all subjects and/or their legal guardian(s).

## Results

### Patient characteristics

Of the 129 AC patients who underwent PTGD, 92 patients did not develop SIRS after PTGD and the remaining 39 patients developed SIRS. In the two groups of patients, gender (female: 50 vs 15, male: 42 vs 22) and age (67.4 vs 65.3) were more evenly distributed, and no statistically significant differences were observed. However, there were statistically significant differences in clinical characteristics such as Blood bacterial, Bile bacteria, DBIL, Obstruction, and Bile properties between the two groups (*p* < 0.05). For AC patients who developed SIRS after PTGD, a higher proportion of bacteria were found in bile culture and peripheral blood culture results. Patients in the SIRS group also had relatively higher DBIL levels at admission (29.0 vs 15.3, *p* = 0.027). A total of 24 out of 39 patients who developed SIRS after PTGD had cystic duct obstruction (64.9%,). In addition, the bile of patients with SIRS was mostly purulent (51.4%). The above results are shown in Table 2.

**Table 2:** Baseline of all patients included in the study.

Variables	no-SIRS (n = 92)	SIRS (n = 37)	*p*
Gender			0.221
Female	50 (54.3%)	15 (40.5%)	
Male	42 (45.7%)	22 (59.5%)	
Age	67.4 (13.8)	65.3 (11.8)	0.399
Blood bacterial			0.03
Gram-negative	5 (5.43%)	6 (16.2%)	
Gram-positive	0 (0.00%)	1 (2.70%)	
None	87 (94.6%)	30 (81.1%)	
Bile bacteria			0.016
Gram-negative	39 (42.4%)	26 (70.3%)	
Gram-positive	4 (4.35%)	0 (0.00%)	
None	49 (53.3%)	11 (29.7%)	
Abdominal pain time	3.44 (3.61)	3.20 (3.95)	0.747
WBC	12.8 (5.54)	14.3 (5.40)	0.164
CRP	141 (103)	128 (70.7)	0.416
DM			0.758
No	76 (82.6%)	29 (78.4%)	
Yes	16 (17.4%)	8 (21.6%)	
Fever			0.241
No	52 (56.5%)	16 (43.2%)	
Yes	40 (43.5%)	21 (56.8%)	
DBIL	15.3 (21.6)	29.0 (34.0)	0.027
Obstruction			0.026
No	54 (58.7%)	13 (35.1%)	
Yes	38 (41.3%)	24 (64.9%)	
Bile properties			0.031
Normal	65 (70.7%)	18 (48.6%)	
Purulent	27 (29.3%)	19 (51.4%)	
Inflammatory exudation			0.578
No	44 (47.8%)	15 (40.5%)	
Yes	48 (52.2%)	22 (59.5%)	
PCT	1.48 (2.18)	5.88 (13.2)	0.051
Length	96.3 (13.9)	102 (19.3)	0.135
Width	45.2 (7.76)	47.1 (11.7)	0.36
Volume factor	4386 (1141)	4748 (1355)	0.157
Wall thickness	5.26 (1.64)	5.39 (1.78)	0.7

### Randomization generated training and validation sets

One hundred and twenty-nine AC patients were divided into training and validation sets at a ratio of 6:4. The baseline of clinical characteristics in the training and validation sets are presented in Tables 3 and 4. In the training set, the results of peripheral blood bacterial culture, fever at admission, and WBC level at the time of hospitalization were statistically different between the two groups (Table 3). However, after the prediction model was constructed, WBC and Blood bacterial were excluded. In the validation set, all clinical characteristics were not statistically significant between the two groups (Table 4).

**Table 3:** Baseline of patients in the training set.

Variables	no-SIRS (n = 53)	SIRS (n = 25)	*p*
Gender			0.522
Female	29 (54.7%)	11 (44.0%)	
Male	24 (45.3%)	14 (56.0%)	
Age	66.5 (14.6)	64.2 (12.5)	0.458
Blood bacterial			0.012
Gram-negative	2 (3.77%)	5 (20.0%)	
Gram-positive	0 (0.00%)	1 (4.00%)	
None	51 (96.2%)	19 (76.0%)	
Bile bacteria			0.066
Gram-negative	21 (39.6%)	17 (68.0%)	
Gram-positive	3 (5.66%)	0 (0.00%)	
None	29 (54.7%)	8 (32.0%)	
Abdominal pain time	3.23 (2.86)	3.39 (4.59)	0.869
WBC	11.6 (5.34)	14.6 (5.82)	0.038
CRP	149 (101)	137 (67.7)	0.541
DM			0.759
No	44 (83.0%)	20 (80.0%)	
Yes	9 (17.0%)	5 (20.0%)	
Fever			0.036
No	32 (60.4%)	8 (32.0%)	
Yes	21 (39.6%)	17 (68.0%)	
DBIL	14.1 (23.4)	25.3 (31.5)	0.122
Obstruction			0.109
No	33 (62.3%)	10 (40.0%)	
Yes	20 (37.7%)	15 (60.0%)	
Bile properties			0.201
Normal	37 (69.8%)	13 (52.0%)	
Purulent	16 (30.2%)	12 (48.0%)	
Inflammatory exudation			0.985
No	25 (47.2%)	11 (44.0%)	
Yes	28 (52.8%)	14 (56.0%)	
PCT	1.32 (1.60)	4.28 (12.8)	0.262
Length	96.0 (12.1)	98.6 (20.8)	0.555
Width	44.7 (7.88)	47.4 (13.4)	0.37
Volume factor	4313 (1041)	4584 (1316)	0.372
Wall thickness	5.30 (1.63)	5.56 (1.76)	0.542

**Table 4:** Baseline of patients in the validation set.

Variables	no-SIRS (n = 39)	SIRS (n = 12)	p
Gender			0.361
Female	21 (53.8%)	4 (33.3%)	
Male	18 (46.2%)	8 (66.7%)	
Age	68.5 (12.8)	67.8 (10.3)	0.84
Blood bacterial			1
Gram-negative	3 (7.69%)	1 (8.33%)	
None	36 (92.3%)	11 (91.7%)	
Bile bacteria			0.258
Gram-negative	18 (46.2%)	9 (75.0%)	
Gram-positive	1 (2.56%)	0 (0.00%)	
None	20 (51.3%)	3 (25.0%)	
Abdominal pain time	3.73 (4.46)	2.79 (2.21)	0.333
WBC	14.5 (5.46)	13.8 (4.58)	0.671
CRP	130 (107)	110 (76.1)	0.459
DM			0.682
No	32 (82.1%)	9 (75.0%)	
Yes	7 (17.9%)	3 (25.0%)	
Fever			0.545
No	20 (51.3%)	8 (66.7%)	
Yes	19 (48.7%)	4 (33.3%)	
DBIL	16.9 (19.2)	36.6 (38.9)	0.114
Obstruction			0.156
No	21 (53.8%)	3 (25.0%)	
Yes	18 (46.2%)	9 (75.0%)	
Bile properties			0.085
Normal	28 (71.8%)	5 (41.7%)	
Purulent	11 (28.2%)	7 (58.3%)	
Inflammatory exudation			0.545
No	19 (48.7%)	4 (33.3%)	
Yes	20 (51.3%)	8 (66.7%)	
PCT	1.69 (2.79)	9.21 (14.0)	0.09
Length	96.7 (16.2)	108 (14.7)	0.038
Width	45.8 (7.63)	46.7 (7.76)	0.748
Volumefactor	4485 (1271)	5089 (1428)	0.207
Wallthickness	5.20 (1.66)	5.04 (1.84)	0.79

### Construction of the prediction model

**Figure 3:**
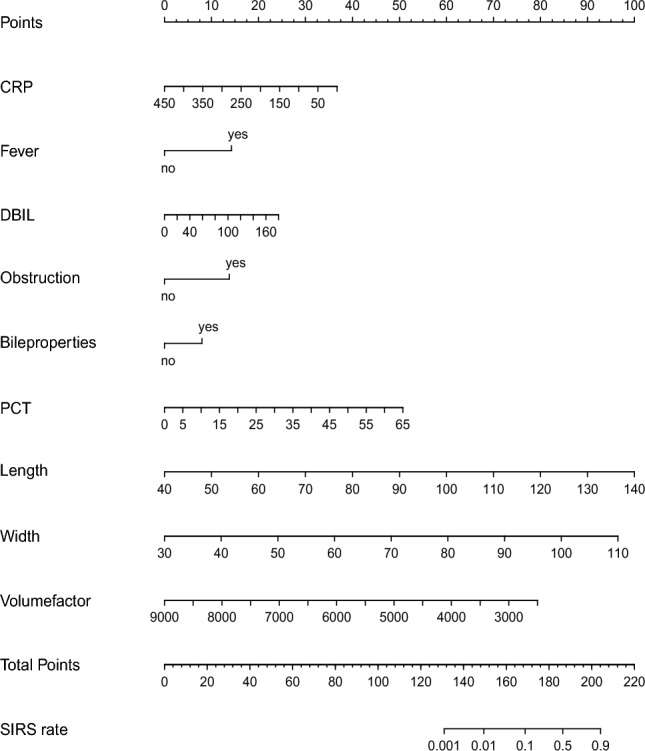
Nomogram of the SIRS prediction model.

All variables in Table 2 were included in the first step of the screening process. Stepwise Regression was used as a method for variable screening. After variable screening was completed, logistic regression was used to construct a model to predict SIRS after PTGD. After these series of analyses, the prediction model was finally constructed (formula = SIRS ~ CRP + Fever + DBIL + Obstruction + Bile properties + PCT + Length + Width + Volume factor). The detailed characteristics of the prediction model are visible in Supplementary Table 1. Most of the variables included in the equation could be objectively measured, with the exception of Obstruction and Bile properties. We present in the Methods section how these two variables are measured, enabling them to be observed relatively objectively. Subsequently, we made a nomogram, and according to the coefficients of different variables in the nomogram, the occurrence probability of SIRS after PTGD could be more intuitively calculated (Fig. 3). The above variable screening and prediction model construction processes were carried out in the training set.

### Evaluation of prediction model

**Figure 4:**
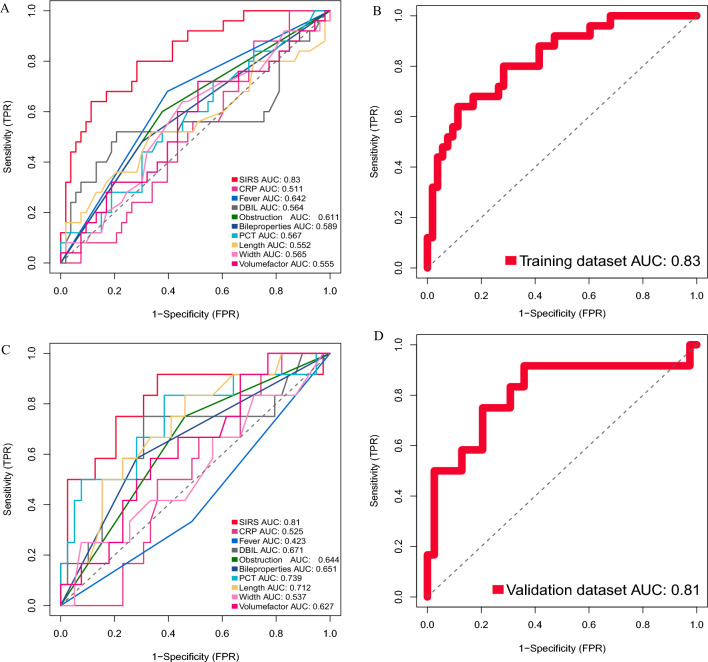
AUC curves for individual clinical features and SIRS prediction model. (**A**) ROC curves for all 9 clinical features and SIRS prediction model in the training set; (**B**) ROC curve for SIRS prediction model in the training set; (**C**) ROC curves for all 9 clinical features and SIRS prediction model in the validation set; (**D**) ROC curve for SIRS prediction model in the validation set.

**Figure 5:**
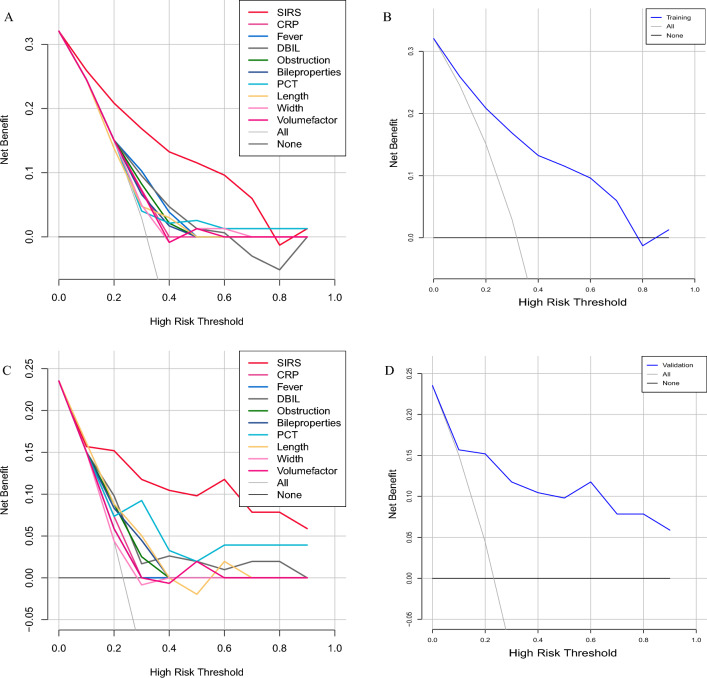
DCA curves for individual clinical features and SIRS prediction model. (**A**) DCA curves for all 9 clinical features and SIRS prediction model in the training set; (**B**) DCA curve for SIRS prediction model in the training set; (**C**) DCA curves for all 9 clinical features and SIRS prediction model in the validation set; (**D**) DCA curve for SIRS prediction model in the validation set.

After the construction of the prediction model, in order to verify the predictive ability of the model, we conducted a validation process in the training set and validation set. The ROC (Receiver operating characteristic) curve was used to evaluate the accuracy of the model prediction. The validation results in the training set showed that the SIRS prediction model we constructed had the largest AUC (Area Under Curve) value, indicating that the prediction accuracy of the model incorporating multiple variables was better than that of the model incorporating a single clinical characteristic (Fig. 4A). Among them, the AUC value of the SIRS prediction model was 0.83 (Fig. 4B), and the AUC values of other models including a single clinical characteristic were smaller than SIRS prediction model (CRP = 0.511, Fever = 0.642, DBIL = 0.564, Obstruction = 0.611, Bile properties = 0.589, PCT = 0.567, Length = 0.552, Width = 0.565, Volume factor = 0.555). DCA (Decision Curve Analysis) was used to further determine the superiority of the SIRS prediction model. Similar to the ROC curve, we also chose to compare the SIRS prediction model with the single clinical characteristic prediction model. The DCA curve showed that the SIRS model had a higher net benefit in predicting the occurrence of SIRS after PTGD if the threshold was within the range of about 8–78% (Fig. 5A, B). Moreover, the net benefit range of SIRS prediction model was larger than that of other prediction models based on single clinical feature (Fig. 5A). These results show that the SIRS prediction model performs well in the training dataset.

Limited by the size of the data, we chose an internal validation method, namely random split validation. The baseline characteristics of the validation set have been shown in Table 4. As shown in Fig. 4C and D, the SIRS prediction model also performed well in the validation set (AUC = 0.81), showing superiority over the model constructed by individual clinical feature (CRP = 0.525, Fever = 0.423, DBIL = 0.671, Obstruction = 0.644, Bile properties = 0.651, PCT = 0.739, Length = 0.712, Width = 0.537, Volume factor = 0.627). In the DCA results, the SIRS prediction model had a net benefit at the approximate threshold of > 18% (Fig. 5C, D). Moreover, the threshold range of net benefit of SIRS prediction model was wider than that of models based on individual clinical characteristic (Fig. 5C). The above results again verified that the SIRS prediction model could accurately predict the probability of SIRS in AC patients after PTGD.

Among the 9 clinical features included in the SIRS prediction model, are there other combinations that can achieve better prediction effect? With such questions, we recombined the 9 clinical features and constructed a total of 511 prediction models, of which NO. 511 was the SIRS prediction model that including all 9 clinical features. The AUCs of these 511 prediction models were calculated in the training and validation sets, respectively, and the results showed that no.511 had the best performance in both datasets (Supplementary Table 2).

## Discussion

The pathophysiological process of AC is thought to arise through three steps^12^. (a) Cholestasis caused by cystic duct obstruction and increased biliary pressure leads to gallbladder mucosal injury and induces a cascade of inflammatory responses. (b) Bacteria invade the gallbladder through the blood and biliary tract. (c) Bacterial proliferation in the gallbladder causes pyogenic infection. The obliteration of the cystic artery or obstruction of the cystic duct causes inflammation in the gallbladder leading to ischemia and necrosis of the gallbladder wall leading to the transformation of AC to gangrenous cholecystitis (GC)^13^. Increased pressure in the gallbladder, release of inflammatory mediators, and release of bacteria or bacterial metabolites into the bloodstream may lead to SIRS^13^.

SRIS are produced by the body in response to stressors such as ischemia and inflammation for defense purposes^14^. SIRS can be divided into two main categories: sepsis and noninfectious inflammation^14^. SIRS in AC similarly includes both types, namely cholecystitis due to bacterial infection and aseptic cholecystitis. However, the dysregulation of the inflammatory cascade can lead to multiple organ dysfunction, resulting in more severe SIRS. These severe complications include organ dysfunction during SIRS, hypoperfusion, hypotension resistant to adequate fluid resuscitation, and even MODS (Multiple Organ Dysfunction Syndrome)^14^. In particular, the effects on the respiratory and circulatory systems lead to the need for intensive care, and also cause the majority of AC-related deaths. Therefore, early identification of SIRS is important for AC patients to receive necessary anti-inflammatory treatment in time, early intensive care intervention, and evaluation of the prognosis of AC patients.

Risk factors for severe cholecystitis include male sex, older age, coronary artery disease, and diabetes mellitus (DM)^15^. In the results of our analysis, the prediction model for SIRS was determined by CRP, Fever, DBIL, Obstruction, Bile properties, Length, Width, and Volume factor. After completing logistic regression analysis, among the 9 clinical characteristic variables, CRP, Fever, Obstruction and Length were associated with SIRS after PTGD (statistically significant). This also indicates that these 4 clinical features are predictors of the severity of cholecystitis. Therefore, this result also provides suggestions for clinicians in the diagnosis and treatment of AC patients with elevated CRP, fever and cystic duct obstruction, more attention should be paid to the possibility of secondary severe gangrenous cholecystitis.

Researchers have been searching for stable methods to predict disease progression in patients with cholecystitis. Some researchers have finally determined NLR and CRP as independent factors for preoperative prediction of gangrenous cholecystitis by analyzing multiple biomarkers (neutrophil to lymphocyte ratio [NLR], C-reactive protein [CRP], platelet to lymphocyte ratio [PLR], lactate and procalcitonin)^16^. With the development of medical technology, the safety of laparoscopic cholecystectomy has been proved, but for the elderly, perioperative complications have always been an important factor affecting the rehabilitation. For older patients, the incidence of serious cardiovascular complications and death correlates with the severity of the septicemic process and inflammation^17^. The presence of necrosis of the gallbladder, inflammatory exudation around the gallbladder, or biliary peritonitis is associated with conversion and open surgery^17^. Indicators related to the degree of gallbladder inflammation, such as cystic duct obstruction, characteristics of bile after percutaneous gallbladder drainage, and inflammatory reaction around the gallbladder, were also included in our study. The results showed that Obstruction and Bile properties were positively correlated with the occurrence of SIRS after PTGD, which also indicates that Obstruction and Bile properties are related to the risk degree of AC. To achieve the prediction of severe cholecystitis, multiple clinical variables (Gallbladder wall thickness, indexes of blood routine examination, including white blood cell, alkaline phosphatase, the percentage of neutrophil, alanine transaminase, aspartate aminotransferase, fibrinogen, gamma-glutamyl transferase, prothrombin time and total bilirubin) were incorporated^18^. After a series of statistical analyses, neutrophil percentage and gallbladder wall thickness were found to be associated with more severe cholecystitis (acute gangrenous cholecystitis, acute purulent cholecystitis)^18^. Similarly, the inflammatory markers CRP and gallbladder wall thickness were also associated with SIRS after PTGD. This also demonstrates again that some objective inflammatory markers and markers related to gallbladder inflammation are sensitive predictors of the severity of the disease during the treatment of cholecystitis.

In this study, we constructed predictive models for the occurrence of SIRS after PTGD across multiple clinical features. Through the use of this model, clinicians can carry out early intervention treatment to prevent SIRS from progressing to MODS and other more serious systemic inflammatory reactions. However, there are some limitations. First, all the patients were from a single center, and external validation data were lacking. Second, there are many clinical features used to construct the model, which brings some difficulties to clinical application. Future studies may collect clinical data from multiple centers, establish multiple external validation datasets, and incorporate clinical characteristics that can be objectively measured as much as possible to construct a model with more accurate prediction and fewer clinical characteristics.

## Conclusions

In summary, we developed a model to predict the occurrence of SIRS after PTGD by clinical characteristics of AC patients. The validation process determined the accuracy of the model in prediction, which provided a reference for clinicians to intervene in time during the treatment of AC to avoid the occurrence of more severe systemic inflammatory response.

### Supplementary Information

Supplementary Table 1.

Supplementary Table 2.

## Data Availability

The datasets created and analyzed for the current investigation are not publically accessible since it would be inconvenient to disclose patient privacy; however, they are available from the corresponding author upon justifiable request (Xuanfeng Zhang: zxfujs@126.com).

## References

[1] Stinton, L. M., Myers, R. P. & Shaffer, E. A. Epidemiology of gallstones. *Gastroenterol. Clin. N. Am.* **39**, 157–169, vii. 10.1016/j.gtc.2010.02.003 (2010). 10.1016/j.gtc.2010.02.003 20478480

[2] Shaffer EA (2006). Gallstone disease: Epidemiology of gallbladder stone disease. Best. Pract. Res. Clin. Gastroenterol..

[3] McSherry CK, Ferstenberg H, Calhoun WF, Lahman E, Virshup M (1985). The natural history of diagnosed gallstone disease in symptomatic and asymptomatic patients. Ann. Surg..

[4] Jin X, Jiang Y, Tang J (2022). Ultrasound-guided percutaneous transhepatic gallbladder drainage improves the prognosis of patients with severe acute cholecystitis. Evid. Based Complement. Altern. Med..

[5] Halldestam I, Enell EL, Kullman E, Borch K (2004). Development of symptoms and complications in individuals with asymptomatic gallstones. Br. J. Surg..

[6] Huber DF, Martin EW, Cooperman M (1983). Cholecystectomy in elderly patients. Am. J. Surg..

[7] Patel N (2018). Interventional radiology-operated cholecystoscopy for the management of symptomatic cholelithiasis: Approach, technical success, safety, and clinical outcomes. AJR Am. J. Roentgenol..

[8] Kang C (2022). The efficacy of percutaneous transhepatic gallbladder drainage combined with gallbladder-preserving cholecystolithotomy in high-risk patients with acute calculous cholecystitis. J. Inflamm. Res..

[9] Stirrat J (2021). Safety and efficacy of percutaneous gallstone extraction in high-risk patients: An alternative to cholecystectomy or long-term drainage?. J. Am. Coll. Surg..

[10] Loftus TJ (2017). Percutaneous cholecystostomy: Prognostic factors and comparison to cholecystectomy. Surg. Endosc..

[11] Cheng Z (2020). The critical roles and mechanisms of immune cell death in sepsis. Front. Immunol..

[12] Kimura Y (2007). Definitions, pathophysiology, and epidemiology of acute cholangitis and cholecystitis: Tokyo Guidelines. J. Hepatobiliary Pancreat. Surg..

[13] Sureka B (2018). Gangrenous cholecystitis: Analysis of imaging findings in histopathologically confirmed cases. Indian J. Radiol. Imaging.

[14] Robertson CM, Coopersmith CM (2006). The systemic inflammatory response syndrome. Microbes Infect..

[15] Tsushimi T (2007). Early laparoscopic cholecystectomy for acute gangrenous cholecystitis. Surg. Laparosc. Endosc. Percutaneous Tech..

[16] Diez Ares JA (2021). Can inflammatory biomarkers help in the diagnosis and prognosis of gangrenous acute cholecystitis? A prospective study. Rev. Esp. Enferm. Dig..

[17] Serban D (2021). Safety of laparoscopic cholecystectomy for acute cholecystitis in the elderly: A multivariate analysis of risk factors for intra and postoperative complications. Medicina (Kaunas).

[18] Chen J, Gao Q, Huang X, Wang Y (2022). Prognostic clinical indexes for prediction of acute gangrenous cholecystitis and acute purulent cholecystitis. BMC Gastroenterol..

