# Supplementary material for: A new method for predicting SIRS after percutaneous transhepatic gallbladder drainage
Source: Sci Rep. 2023 Dec 6;13:21523. doi: 10.1038/s41598-023-48908-6 (PMC10700562; doi:10.1038/s41598-023-48908-6)
Supplement: Supplementary file 1 — Supplementary Table 1. [file 41598_2023_48908_MOESM1_ESM.pdf]

characteristics	coefficients	p
(Intercept)	-14.42737	0.00931
CRP	-0.010146	0.0259
Fever	1.77001	0.01659
DBIL	0.016772	0.11382
Obstruction	1.712263	0.01501
Bileproperties	0.990431	0.11997
PCT	0.09703	0.21848
Length	0.124331	0.03711
Width	0.149942	0.08547
Volumefactor	-0.001518	0.13211

SupplementaryTable1: SIRS prediction model.
