# Supplementary material for: A new method for predicting SIRS after percutaneous transhepatic gallbladder drainage
Source: Sci Rep. 2023 Dec 6;13:21523. doi: 10.1038/s41598-023-48908-6 (PMC10700562; doi:10.1038/s41598-023-48908-6)
Supplement: Supplementary file 2 — Supplementary Table 2. [file 41598_2023_48908_MOESM2_ESM.pdf]

training set			validation set		
model	auc	n.characteristics	model	auc	n.characteristics
511	0.830189	9	511	0.80982906	9
506	0.818868	8	506	0.777777778	8
502	0.817358	8	502	0.775641026	8
505	0.806792	8	505	0.722222222	8
504	0.803774	8	504	0.764957265	8
508	0.803774	8	508	0.769230769	8
468	0.803019	7	468	0.762820513	7
466	0.800755	7	466	0.784188034	7
472	0.800755	7	472	0.762820513	7
503	0.800755	8	503	0.77991453	8
510	0.798491	8	510	0.807692308	8
473	0.794717	7	473	0.756410256	7
467	0.793962	7	467	0.767094017	7
494	0.793962	7	494	0.771367521	7
382	0.792453	6	382	0.752136752	6
469	0.792453	7	469	0.683760684	7
386	0.791698	6	386	0.760683761	6
475	0.791698	7	475	0.702991453	7
495	0.790943	7	495	0.773504274	7
383	0.790189	6	383	0.700854701	6
497	0.790189	7	497	0.711538462	7
438	0.788679	6	438	0.771367521	6
388	0.78717	6	388	0.739316239	6
474	0.78717	7	474	0.741452991	7
485	0.78717	7	485	0.717948718	7
509	0.78717	8	509	0.856837607	8
481	0.786415	7	481	0.737179487	7
507	0.784906	8	507	0.826923077	8
470	0.784151	7	470	0.69017094	7
385	0.783396	6	385	0.675213675	6
440	0.783396	6	440	0.75	6
444	0.783396	6	444	0.75	6
471	0.783396	7	471	0.677350427	7
441	0.782642	6	441	0.698717949	6
404	0.781887	6	404	0.72008547	6
484	0.781887	7	484	0.662393162	7
496	0.781887	7	496	0.752136752	7
498	0.781887	7	498	0.773504274	7
408	0.781132	6	408	0.711538462	6
483	0.780377	7	483	0.717948718	7
445	0.778868	6	445	0.741452991	6
389	0.778113	6	389	0.685897436	6
332	0.777358	5	332	0.717948718	5
258	0.776604	5	258	0.677350427	5
447	0.776604	6	447	0.696581197	6
442	0.775849	6	442	0.692307692	6
482	0.775849	7	482	0.737179487	7
327	0.775094	5	327	0.681623932	5
402	0.775094	6	402	0.743589744	6
501	0.775094	7	501	0.852564103	7
390	0.77434	6	390	0.670940171	6
329	0.773585	5	329	0.670940171	5
446	0.773585	6	446	0.722222222	6
333	0.77283	5	333	0.670940171	5
334	0.77283	5	334	0.673076923	5
443	0.77283	6	443	0.673076923	6

439	0.772075	6	439	0.752136752	6
260	0.771321	5	260	0.658119658	5
330	0.771321	5	330	0.752136752	5
190	0.770566	4	190	0.655982906	4
271	0.770566	5	271	0.72008547	5
476	0.770566	7	476	0.784188034	7
391	0.769811	6	391	0.655982906	6
188	0.769057	4	188	0.673076923	4
335	0.769057	5	335	0.662393162	5
387	0.769057	6	387	0.72008547	6
405	0.768302	6	405	0.645299145	6
411	0.768302	6	411	0.632478632	6
384	0.767547	6	384	0.653846154	6
403	0.767547	6	403	0.709401709	6
277	0.766792	5	277	0.69017094	5
326	0.766792	5	326	0.739316239	5
409	0.766038	6	409	0.715811966	6
491	0.766038	7	491	0.831196581	7
406	0.765283	6	406	0.647435897	6
500	0.765283	7	500	0.752136752	7
479	0.764528	7	479	0.688034188	7
492	0.764528	7	492	0.83974359	7
410	0.763019	6	410	0.700854701	6
489	0.763019	7	489	0.831196581	7
256	0.762264	5	256	0.658119658	5
418	0.762264	6	418	0.835470085	6
453	0.762264	6	453	0.730769231	6
487	0.762264	7	487	0.841880342	7
275	0.761509	5	275	0.711538462	5
462	0.761509	6	462	0.784188034	6
257	0.760755	5	257	0.715811966	5
499	0.76	7	499	0.80982906	7
272	0.759245	5	272	0.655982906	5
278	0.759245	5	278	0.611111111	5
274	0.758491	5	274	0.634615385	5
459	0.758491	6	459	0.826923077	6
398	0.757736	6	398	0.754273504	6
348	0.756981	5	348	0.705128205	5
361	0.756981	5	361	0.841880342	5
460	0.756981	6	460	0.83974359	6
454	0.756226	6	454	0.728632479	6
455	0.756226	6	455	0.702991453	6
456	0.756226	6	456	0.64957265	6
279	0.755472	5	279	0.598290598	5
328	0.755472	5	328	0.66025641	5
419	0.755472	6	419	0.837606838	6
488	0.755472	7	488	0.841880342	7
407	0.754717	6	407	0.632478632	6
478	0.754717	7	478	0.754273504	7
346	0.753962	5	346	0.72008547	5
352	0.753962	5	352	0.683760684	5
463	0.753962	6	463	0.824786325	6
486	0.753962	7	486	0.803418803	7
490	0.753962	7	490	0.792735043	7
136	0.753208	4	136	0.621794872	4
349	0.753208	5	349	0.636752137	5
417	0.753208	6	417	0.841880342	6
493	0.752453	7	493	0.837606838	7

259	0.751698	5	259	0.61965812	5
276	0.751698	5	276	0.666666667	5
280	0.751698	5	280	0.581196581	5
395	0.751698	6	395	0.645299145	6
138	0.750189	4	138	0.611111111	4
140	0.750189	4	140	0.583333333	4
451	0.750189	6	451	0.666666667	6
137	0.749434	4	137	0.658119658	4
331	0.749434	5	331	0.705128205	5
423	0.749434	6	423	0.824786325	6
425	0.749434	6	425	0.820512821	6
480	0.749434	7	480	0.790598291	7
273	0.748679	5	273	0.617521368	5
457	0.748679	6	457	0.69017094	6
362	0.747925	5	362	0.822649573	5
392	0.747925	6	392	0.794871795	6
350	0.74717	5	350	0.628205128	5
477	0.74717	7	477	0.786324786	7
412	0.746415	6	412	0.760683761	6
426	0.746415	6	426	0.773504274	6
448	0.746415	6	448	0.760683761	6
291	0.74566	5	291	0.837606838	5
295	0.74566	5	295	0.831196581	5
363	0.74566	5	363	0.814102564	5
367	0.74566	5	367	0.811965812	5
394	0.74566	6	394	0.762820513	6
186	0.744906	4	186	0.655982906	4
354	0.744906	5	354	0.66025641	5
399	0.744906	6	399	0.752136752	6
208	0.744151	4	208	0.598290598	4
351	0.744151	5	351	0.617521368	5
400	0.744151	6	400	0.715811966	6
204	0.743396	4	204	0.615384615	4
424	0.743396	6	424	0.829059829	6
207	0.742642	4	207	0.658119658	4
267	0.742642	5	267	0.726495726	5
353	0.742642	5	353	0.679487179	5
355	0.742642	5	355	0.617521368	5
210	0.741887	4	210	0.574786325	4
297	0.741887	5	297	0.822649573	5
461	0.741887	6	461	0.814102564	6
464	0.741887	6	464	0.835470085	6
225	0.741132	4	225	0.820512821	4
189	0.740377	4	189	0.626068376	4
415	0.740377	6	415	0.658119658	6
130	0.739623	4	130	0.615384615	4
287	0.739623	5	287	0.700854701	5
364	0.739623	5	364	0.764957265	5
84	0.738868	3	84	0.566239316	3
397	0.738868	6	397	0.602564103	6
202	0.738113	4	202	0.632478632	4
339	0.738113	5	339	0.615384615	5
369	0.738113	5	369	0.799145299	5
393	0.738113	6	393	0.758547009	6
420	0.737358	6	420	0.777777778	6
347	0.736981	5	347	0.675213675	5
209	0.736604	4	209	0.587606838	4
368	0.736604	5	368	0.816239316	5

396	0.736604	6	396	0.628205128	6
262	0.735849	5	262	0.643162393	5
264	0.735849	5	264	0.608974359	5
370	0.735849	5	370	0.777777778	5
432	0.735849	6	432	0.822649573	6
221	0.735094	4	221	0.835470085	4
421	0.735094	6	421	0.782051282	6
427	0.735094	6	427	0.820512821	6
227	0.73434	4	227	0.803418803	4
312	0.73434	5	312	0.818376068	5
449	0.73434	6	449	0.769230769	6
205	0.733585	4	205	0.677350427	4
268	0.733585	5	268	0.602564103	5
284	0.733585	5	284	0.611111111	5
338	0.733585	5	338	0.739316239	5
401	0.733585	6	401	0.645299145	6
416	0.733585	6	416	0.728632479	6
203	0.733208	4	203	0.581196581	4
47	0.73283	3	47	0.532051282	3
265	0.73283	5	265	0.764957265	5
301	0.73283	5	301	0.841880342	5
341	0.73283	5	341	0.596153846	5
450	0.73283	6	450	0.732905983	6
187	0.732075	4	187	0.707264957	4
289	0.732075	5	289	0.664529915	5
336	0.732075	5	336	0.77991453	5
337	0.732075	5	337	0.724358974	5
434	0.732075	6	434	0.820512821	6
302	0.731321	5	302	0.816239316	5
340	0.731321	5	340	0.611111111	5
345	0.731321	5	345	0.634615385	5
139	0.730566	4	139	0.457264957	4
414	0.730566	6	414	0.737179487	6
286	0.729811	5	286	0.572649573	5
296	0.729811	5	296	0.818376068	5
80	0.729434	3	80	0.581196581	3
194	0.729057	4	194	0.594017094	4
228	0.729057	4	228	0.756410256	4
342	0.729057	5	342	0.722222222	5
222	0.728302	4	222	0.77991453	4
365	0.728302	5	365	0.769230769	5
82	0.727925	3	82	0.605769231	3
269	0.727547	5	269	0.585470085	5
366	0.727547	5	366	0.760683761	5
422	0.727547	6	422	0.767094017	6
452	0.727547	6	452	0.767094017	6
148	0.726792	4	148	0.544871795	4
198	0.726792	4	198	0.587606838	4
224	0.726792	4	224	0.762820513	4
135	0.726038	4	135	0.570512821	4
147	0.726038	4	147	0.677350427	4
270	0.726038	5	270	0.561965812	5
429	0.726038	6	429	0.811965812	6
201	0.72566	4	201	0.679487179	4
192	0.725283	4	192	0.621794872	4
193	0.725283	4	193	0.579059829	4
266	0.725283	5	266	0.698717949	5
263	0.724528	5	263	0.587606838	5

283	0.724528	5	283	0.730769231	5
290	0.724528	5	290	0.585470085	5
293	0.724528	5	293	0.762820513	5
313	0.724528	5	313	0.824786325	5
150	0.723774	4	150	0.510683761	4
226	0.723774	4	226	0.803418803	4
294	0.723774	5	294	0.773504274	5
298	0.723774	5	298	0.760683761	5
261	0.723019	5	261	0.764957265	5
458	0.723019	6	458	0.790598291	6
97	0.722264	3	97	0.784188034	3
133	0.722264	4	133	0.604700855	4
292	0.722264	5	292	0.790598291	5
371	0.722264	5	371	0.799145299	5
144	0.721509	4	144	0.585470085	4
413	0.721509	6	413	0.754273504	6
223	0.720755	4	223	0.764957265	4
199	0.72	4	199	0.570512821	4
431	0.72	6	431	0.818376068	6
288	0.719245	5	288	0.700854701	5
343	0.719245	5	343	0.722222222	5
465	0.719245	6	465	0.831196581	6
83	0.718491	3	83	0.472222222	3
134	0.718491	4	134	0.527777778	4
153	0.717736	4	153	0.792735043	4
195	0.717736	4	195	0.737179487	4
285	0.717736	5	285	0.594017094	5
428	0.717736	6	428	0.833333333	6
200	0.716981	4	200	0.452991453	4
74	0.716604	3	74	0.641025641	3
191	0.716226	4	191	0.752136752	4
282	0.716226	5	282	0.741452991	5
52	0.715472	3	52	0.514957265	3
155	0.715472	4	155	0.756410256	4
299	0.715472	5	299	0.777777778	5
344	0.715472	5	344	0.683760684	5
356	0.715472	5	356	0.739316239	5
433	0.715472	6	433	0.829059829	6
99	0.714717	3	99	0.743589744	3
149	0.714717	4	149	0.525641026	4
281	0.714717	5	281	0.767094017	5
300	0.714717	5	300	0.747863248	5
359	0.714717	5	359	0.636752137	5
374	0.714717	5	374	0.715811966	5
242	0.71434	4	242	0.799145299	4
152	0.713962	4	152	0.826923077	4
197	0.713962	4	197	0.692307692	4
230	0.713962	4	230	0.737179487	4
311	0.713208	5	311	0.835470085	5
437	0.713208	6	437	0.833333333	6
151	0.712453	4	151	0.792735043	4
358	0.712453	5	358	0.707264957	5
77	0.711698	3	77	0.572649573	3
79	0.711698	3	79	0.549145299	3
373	0.711698	5	373	0.790598291	5
430	0.711698	6	430	0.741452991	6
232	0.710943	4	232	0.790598291	4
303	0.710943	5	303	0.822649573	5

435	0.710943	6	435	0.764957265	6
229	0.710189	4	229	0.762820513	4
436	0.710189	6	436	0.788461538	6
306	0.709434	5	306	0.688034188	5
131	0.708679	4	131	0.591880342	4
143	0.708679	4	143	0.544871795	4
376	0.708679	5	376	0.80982906	5
304	0.707925	5	304	0.705128205	5
145	0.70717	4	145	0.713675214	4
206	0.706792	4	206	0.658119658	4
132	0.706415	4	132	0.696581197	4
214	0.706415	4	214	0.585470085	4
19	0.70566	2	19	0.504273504	2
166	0.70566	4	166	0.807692308	4
95	0.704906	3	95	0.773504274	3
213	0.704906	4	213	0.713675214	4
377	0.704151	5	377	0.811965812	5
378	0.704151	5	378	0.801282051	5
212	0.703774	4	212	0.705128205	4
142	0.703396	4	142	0.617521368	4
146	0.703396	4	146	0.655982906	4
196	0.703396	4	196	0.66025641	4
233	0.703396	4	233	0.807692308	4
243	0.703396	4	243	0.803418803	4
168	0.702642	4	168	0.728632479	4
141	0.701887	4	141	0.735042735	4
96	0.701132	3	96	0.811965812	3
216	0.701132	4	216	0.547008547	4
317	0.701132	5	317	0.777777778	5
357	0.701132	5	357	0.728632479	5
372	0.701132	5	372	0.816239316	5
156	0.700377	4	156	0.818376068	4
87	0.7	3	87	0.483974359	3
51	0.699623	3	51	0.549145299	3
75	0.699623	3	75	0.442307692	3
375	0.699623	5	375	0.811965812	5
379	0.699623	5	379	0.768162393	5
158	0.698113	4	158	0.673076923	4
231	0.697358	4	231	0.822649573	4
307	0.697358	5	307	0.803418803	5
78	0.696604	3	78	0.478632479	3
217	0.696604	4	217	0.664529915	4
241	0.695849	4	241	0.816239316	4
46	0.695094	3	46	0.480769231	3
50	0.695094	3	50	0.467948718	3
98	0.695094	3	98	0.728632479	3
234	0.695094	4	234	0.685897436	4
236	0.695094	4	236	0.677350427	4
88	0.69434	3	88	0.547008547	3
316	0.69434	5	316	0.739316239	5
92	0.693585	3	92	0.474358974	3
154	0.693585	4	154	0.75	4
314	0.69283	5	314	0.747863248	5
112	0.692453	3	112	0.722222222	3
211	0.692075	4	211	0.739316239	4
310	0.692075	5	310	0.72008547	5
100	0.691321	3	100	0.805555556	3
110	0.691321	3	110	0.769230769	3

157	0.691321	4	157	0.739316239	4
219	0.691321	4	219	0.621794872	4
48	0.690566	3	48	0.536324786	3
160	0.690566	4	160	0.824786325	4
246	0.690566	4	246	0.727564103	4
380	0.690566	5	380	0.788461538	5
81	0.690189	3	81	0.658119658	3
215	0.689057	4	215	0.568376068	4
102	0.688302	3	102	0.673076923	3
240	0.688302	4	240	0.711538462	4
244	0.688302	4	244	0.75534188	4
220	0.687547	4	220	0.555555556	4
319	0.687547	5	319	0.771367521	5
360	0.687547	5	360	0.700854701	5
49	0.686792	3	49	0.634615385	3
94	0.686792	3	94	0.506410256	3
169	0.686792	4	169	0.739316239	4
318	0.686792	5	318	0.777777778	5
76	0.685283	3	76	0.675213675	3
308	0.685283	5	308	0.816239316	5
24	0.683774	2	24	0.506410256	2
113	0.683774	3	113	0.74465812	3
170	0.683774	4	170	0.788461538	4
315	0.683774	5	315	0.747863248	5
323	0.683774	5	323	0.797008547	5
93	0.683019	3	93	0.489316239	3
90	0.682642	3	90	0.39957265	3
53	0.682264	3	53	0.784188034	3
237	0.682264	4	237	0.77991453	4
177	0.681509	4	177	0.794871795	4
218	0.681509	4	218	0.645299145	4
321	0.681509	5	321	0.805555556	5
91	0.680755	3	91	0.621794872	3
309	0.680755	5	309	0.788461538	5
172	0.68	4	172	0.769230769	4
159	0.678491	4	159	0.711538462	4
245	0.678491	4	245	0.751068376	4
162	0.677736	4	162	0.801282051	4
86	0.677358	3	86	0.569444444	3
103	0.676981	3	103	0.69017094	3
247	0.676981	4	247	0.775641026	4
101	0.675472	3	101	0.715811966	3
235	0.675472	4	235	0.692307692	4
10	0.674717	2	10	0.583333333	2
20	0.674717	2	20	0.502136752	2
171	0.674717	4	171	0.75	4
305	0.674717	5	305	0.709401709	5
85	0.673208	3	85	0.69017094	3
163	0.673208	4	163	0.677350427	4
239	0.673208	4	239	0.771367521	4
22	0.67283	2	22	0.471153846	2
249	0.672453	4	249	0.769230769	4
111	0.672075	3	111	0.746794872	3
89	0.671698	3	89	0.66025641	3
167	0.671698	4	167	0.762820513	4
381	0.671698	5	381	0.824786325	5
23	0.670943	2	23	0.568376068	2
106	0.670189	3	106	0.77991453	3

107	0.668679	3	107	0.658119658	3
161	0.667925	4	161	0.784188034	4
115	0.667547	3	115	0.756410256	3
248	0.66717	4	248	0.775641026	4
25	0.66566	2	25	0.760683761	2
114	0.66566	3	114	0.782051282	3
165	0.66566	4	165	0.658119658	4
250	0.66566	4	250	0.72542735	4
56	0.664906	3	56	0.741452991	3
104	0.664906	3	104	0.797008547	3
116	0.663396	3	116	0.769230769	3
59	0.662642	3	59	0.72008547	3
238	0.662642	4	238	0.790598291	4
28	0.661887	2	28	0.717948718	2
176	0.661887	4	176	0.822649573	4
324	0.661887	5	324	0.732905983	5
18	0.659623	2	18	0.448717949	2
54	0.658868	3	54	0.715811966	3
320	0.658868	5	320	0.715811966	5
121	0.658491	3	121	0.790598291	3
117	0.658113	3	117	0.710470085	3
105	0.657358	3	105	0.762820513	3
109	0.657358	3	109	0.636752137	3
119	0.656604	3	119	0.693376068	3
31	0.655849	2	31	0.705128205	2
251	0.655849	4	251	0.805555556	4
32	0.655094	2	32	0.754273504	2
58	0.655094	3	58	0.673076923	3
173	0.65434	4	173	0.692307692	4
174	0.653585	4	174	0.694444444	4
175	0.653585	4	175	0.683760684	4
178	0.653585	4	178	0.801282051	4
108	0.65283	3	108	0.664529915	3
60	0.651321	3	60	0.732905983	3
179	0.651321	4	179	0.685897436	4
322	0.651321	5	322	0.80982906	5
30	0.649811	2	30	0.653846154	2
164	0.649811	4	164	0.683760684	4
253	0.649811	4	253	0.797008547	4
35	0.649057	2	35	0.701923077	2
118	0.649057	3	118	0.706196581	3
181	0.648302	4	181	0.658119658	4
26	0.647925	2	26	0.701923077	2
62	0.647547	3	62	0.675213675	3
63	0.647547	3	63	0.69017094	3
66	0.646792	3	66	0.645299145	3
33	0.64566	2	33	0.721153846	2
21	0.645283	2	21	0.594017094	2
38	0.643774	2	38	0.633547009	2
61	0.643774	3	61	0.702991453	3
57	0.643019	3	57	0.647435897	3
27	0.642264	2	27	0.788461538	2
64	0.642264	3	64	0.797008547	3
125	0.642264	3	125	0.650641026	3
2	0.641887	1	2	0.423076923	1
55	0.641509	3	55	0.790598291	3
254	0.641509	4	254	0.733974359	4
34	0.640755	2	34	0.672008547	2

325	0.637736	5	325	0.784188034	5
29	0.636981	2	29	0.61965812	2
122	0.635472	3	122	0.807692308	3
182	0.633962	4	182	0.769230769	4
120	0.633208	3	120	0.811965812	3
123	0.633208	3	123	0.676282051	3
36	0.63283	2	36	0.786324786	2
252	0.630943	4	252	0.811965812	4
180	0.621132	4	180	0.69017094	4
65	0.620377	3	65	0.713675214	3
67	0.620377	3	67	0.688034188	3
12	0.618113	2	12	0.64957265	2
39	0.615849	2	39	0.682692308	2
68	0.61283	3	68	0.773504274	3
124	0.612075	3	124	0.686965812	3
183	0.612075	4	183	0.769230769	4
4	0.611321	1	4	0.644230769	1
184	0.609057	4	184	0.745726496	4
11	0.607547	2	11	0.717948718	2
69	0.600755	3	69	0.728632479	3
37	0.598868	2	37	0.71474359	2
126	0.598491	3	126	0.767094017	3
185	0.598491	4	185	0.681623932	4
41	0.597358	2	41	0.743589744	2
71	0.596981	3	71	0.626068376	3
255	0.596981	4	255	0.801282051	4
70	0.594717	3	70	0.758547009	3
13	0.590189	2	13	0.655982906	2
5	0.589057	1	5	0.650641026	1
128	0.587925	3	128	0.756410256	3
15	0.586415	2	15	0.679487179	2
73	0.584906	3	73	0.585470085	3
16	0.583396	2	16	0.452991453	2
129	0.581887	3	129	0.697649573	3
45	0.580377	2	45	0.590811966	2
42	0.577358	2	42	0.775641026	2
43	0.577358	2	43	0.625	2
40	0.573585	2	40	0.790598291	2
127	0.568302	3	127	0.775641026	3
6	0.56566	1	6	0.739316239	1
72	0.565283	3	72	0.632478632	3
8	0.564906	1	8	0.537393162	1
17	0.564528	2	17	0.63034188	2
3	0.563774	1	3	0.670940171	1
14	0.560755	2	14	0.709401709	2
9	0.555472	1	9	0.627136752	1
7	0.552453	1	7	0.711538462	1
44	0.550189	2	44	0.625	2
1	0.510943	1	1	0.523504274	1

SupplementaryTable2: 511 prediction models including 9 clinical characteristics.
